# A patient with respiratory toxigenic diphtheria in Greece after more than 30 years

**DOI:** 10.1017/S0950268820002605

**Published:** 2020-10-28

**Authors:** T. Georgakopoulou, K. Tryfinopoulou, A. Doudoulakakis, F. Nikolaou, I. Magaziotou, A. Flountzi, N. K. Fry, D. J Litt, M. Damala, I. Spiliopoulou, E. Liatsi-Douvitsa, E. Lebessi, G. Panayiotakopoulos, M. Tsolia, G. Saroglou, M. Theodoridou, S. Tsiodras, A. Efstratiou

**Affiliations:** 1 Department for Vaccine Preventable Diseases & Congenital Diseases, National Public Health Organization (NPHO), Athens, Greece; 2 Central Public Health Laboratory, National Public Health Organization (NPHO), Athens, Greece; 3 Department of Microbiology, P. & A. Kyriakou Children's Hospital, Athens, Greece; 4 Paediatric Intensive Care Unit, P. & A. Kyriakou Children's Hospital, Athens, Greece; 5 WHO Global Collaborating Centre for Reference and Research on Diphtheria and Streptococcal Infections, Reference Microbiology Division, National Infection Service, Public Health England, London, UK; 6 Immunisation and Countermeasures Division, Public Health England – National Infection Service, London, UK; 7 Research Unit of Advanced Composite Nano Materials & Nanotechnology, School of Chemical Engineering, National Technical University of Athens, Athens, Greece; 8 National Public Health Organization (NPHO), Athens, Greece; 9 2nd Department of Paediatrics, National and Kapodistrian University of Athens School of Health Sciences, Greece; 10 Internal Medicine Department, Metropolitan Hospital, Athens, Greece; 11 First Department of Pediatrics, Aghia Sophia Children's Hospital, University of Athens, Greece; 12 4th Department of Internal Medicine, Attikon University Hospital, Medical School, National and Kapodistrian University of Athens, Greece

**Keywords:** Diphtheria, DAT, epidemiological surveillance, diphtheria vaccination, Greece

## Abstract

The introduction of treatment and systematic vaccination has significantly reduced diphtheria mortality; however, toxigenic strains continue to circulate worldwide. The emergence of an indigenous diphtheria case with fatal outcome in Greece, after 30 years, raised challenges for laboratory confirmation, clinical and public health management. Toxigenic *Corynebacterium diphtheriae* was isolated from an incompletely vaccinated 8-year-old boy with underlying conditions. The child passed away due to respiratory distress syndrome, before the administration of diphtheria antitoxin (DAT). All close contacts in family, school and hospital settings were investigated. Pharyngeal swabs were obtained to determine asymptomatic carriage. Chemoprophylaxis was given for 7 days to all close contacts and a booster dose to those incompletely vaccinated. Testing revealed a classmate, belonging to a subpopulation group (Roma), and incompletely vaccinated, as an asymptomatic carrier with an indistinguishable toxigenic strain (same novel multilocus sequence type, designated ST698). This case highlights the role of asymptomatic carriage, as the entry of toxigenic strains into susceptible populations can put individuals and their environment at risk. Maintenance of high-level epidemiological and microbiological surveillance, implementation of systematic vaccination in children and adults with primary and booster doses, availability of a DAT stockpile, and allowing timely administration are the cornerstone to prevent similar incidents in the future.

## Introduction

Diphtheria is a vaccine-preventable infectious disease caused by exotoxin producing *Corynebacterium* species: mainly *Corynebacterium diphtheriae* and *Corynebacterium ulcerans* and occasionally *Corynebacterium pseudotuberculosis* [1]. It affects mainly the mucous membranes of the upper respiratory tract (nose, pharynx, larynx, tonsils and trachea) and less frequently the skin and mucous membranes at other sites [2,3]. The disease spreads from person-to-person via droplets and direct contact with respiratory secretions and rarely from skin lesions and fomites [2]. The incubation period is 2–5 days but can reach 10 days [4,5]. Humans are thought to be the major reservoir for toxigenic *C. diphtheriae* [4], but isolation from equine wounds has been reported [6]. In contrast, toxigenic *C. ulcerans* has been isolated from a wide range of animals including companion animals and is considered a zoonotic species. *C. pseudotuberculosis* is the causative agent of caseous lymphadenitis, a disease common in small ruminant populations, and which is potentially zoonotic, although reports of human infection are rare [7].

In the pre-vaccination era, diphtheria was a common cause of morbidity and mortality. The introduction of mass infant immunisation programmes changed the epidemiology of the disease and a significant reduction in the incidence was observed in Europe [8–10]. However, the re-emergence of diphtheria in the Newly Independent States of the former Soviet Union in the 1990s led to the report of more than 98 000 cases and 3400 deaths during the peak of the epidemic (1994–1995) [11]. In recent years, only sporadic cases of diphtheria have been reported in the European Union (EU)/European Economic Area (EEA), most of which were cutaneous (only 4 of 17 cases with toxigenic *C. diphtheriae*, with known clinical presentation, were classical respiratory diphtheria). The majority of these cases are imported from endemic areas and the increased number of susceptible travellers probably conduced to the distribution. A total of 18 *C. diphtheriae* and 21 *C. ulcerans* cases were reported in 2017 with an overall notification rate below 0.01/100 000 population. Latvia is the sole country in the EU/EEA still reporting indigenous *C. diphtheriae* cases since 2012, which are attributable to the regional epidemic in the 1990s [1]. However, there has recently been a resurgence of the disease in many regions of the world, mainly due to the social unrest and movement of populations combined with the breakdown of immunisation programmes, for example in Bangladesh, Yemen and Syria [12–14].

Classic respiratory diphtheria has a gradual onset with the development of a sore throat, low-grade fever and mild exudative pharyngitis. Pseudomembranes, thick greyish membranes firmly attached to the underlying mucosa, if present usually starts forming after 2–3 days. In severe cases, enlarged anterior cervical lymph nodes and oedema of the surrounding tissue produce a characteristic ‘bull-neck’ appearance [2,5,15]. The toxin produced has a preference for the myocardium and the nervous system [16]. Most infections in highly vaccinated populations are asymptomatic or result in a mild clinical course [17]. Diphtheria is fatal in 5–10% of cases with a higher mortality rate in children younger than 5 and adults over 40 years of age [4]. Successful treatment depends on the rapid administration of diphtheria antitoxin (DAT) in combination with antibiotics (macrolides or benzylpenicillin).

Asymptomatic respiratory carriage is important in sustaining diphtheria while lack of immunity may increase susceptibility to colonisation by toxigenic corynebacteria in the nasopharynx [18,19]. Evidence exists that cutaneous diphtheria may be more transmissible than respiratory diphtheria and, in tropical countries, cutaneous diphtheria lesions may act as reservoirs of infection [15,20,21].

Laboratory confirmation of the diagnosis is crucial for timely and appropriate public health management of re-emerging diphtheria cases. Laboratory diagnosis in the clinical laboratory is by culture on blood agar and tellurite-containing media and microscopic examination of suspicious colonies. This is typically followed by analysis using commercial systems, e.g. API® Coryne, VITEK® microbial identification system (bioMérieux SA, Marcy-l'Etoile, France) or matrix-assisted laser desorption/ionisation time of flight mass spectrometry (MALDI-TOF MS) to putatively identify as either *C. diphtheriae*, *C. ulcerans* or *C. pseudotuberculosis*. However, the confirmation of identification and the determination of toxigenicity of potentially toxigenic corynebacteria in Europe usually require submission to a national reference laboratory or the World Health Organisation (WHO) Collaborating Centre for Diphtheria in the UK for further analysis including the Elek test for diphtheria toxin production [21].

Diphtheria vaccines are recommended for both infants, children, teens and adults. A single-antigen diphtheria toxoid is not available and is usually found in combination with tetanus toxoid as paediatric diphtheria–tetanus toxoid (DT) or adult tetanus–diphtheria (Td), and with both tetanus toxoid and acellular pertussis vaccine as DTaP and Tdap. Paediatric formulations (DT and DTaP) contain a similar amount of tetanus toxoid as adult Td but contain 3–4 times as much diphtheria toxoid. Combination vaccines that include Hepatitis B, Poliomyelitis and Haemophilus influenza type b are also available [4].

Diphtheria is a notifiable disease in Greece and surveillance is performed through the mandatory notification system using the European Commission's case definition as amended in 2012 (Decision 2012/506EU).

Herein, we describe an indigenous case of diphtheria from Greece; we present the epidemiological and microbiological investigations as well as the potential public health implications.

## Case

The case was an 8-year-old boy with underlying conditions (mosaic Down syndrome (DS) with pulmonary hypertension) who had been incompletely vaccinated with three doses of DTaP vaccine in infancy.

On day 1 (in November 2019), the patient presented high fever (39.9 °C) and later on during the night respiratory distress. The child was taken to a local hospital on day 2 where laryngitis was diagnosed and inhaled adrenaline was administered in the emergency department. However, his condition did not show improvement and was transferred to the paediatric intensive care unit of a paediatric reference hospital where he was intubated. Upon admission, the boy was pale, with diminished breath sounds, respiratory stridor and wheezing, abdominal breathing with intercostal retractions and abnormal vital signs: sat O_2_ <90%, respiration rate = 60/min, heart rate = 150/min and blood pressure = 110/60 mmHg. The physical examination of the pharynx revealed only some minor exudate patches. He initially received ceftriaxone and on day 3 vancomycin (250 mg q.i.d. I.V.) was added due to the radiological findings of pulmonary consolidation in the left lower lobe, increased inflammatory markers, haemodynamic instability and increased respiratory needs. During hospitalisation he developed fever up to 38.5 °C, while the white blood cell count increased to 34 600/cm^3^ and the CRP (C-reactive protein) up to 93 mg/L.

A Gram stain of the bronchial secretions revealed many Gram (+) coryneform bacteria and polymorphonuclear leucocytes. On Filmarray® respiratory panel (BioFire Diagnostics, Salt Lake City, USA) rhinovirus/enterovirus was detected and while waiting for *Pneumocystis jirovecii* polymerase chain reaction (PCR) on bronchoalveolar lavage, trimethoprim-sulphomethoxazole was started (day 5). On day 6, being severely hypoxemic, haemodynamically unstable, in profound metabolic acidosis the child had a cardiac arrest and passed away. On the same day, the Gram (+) bacterium of bronchial secretions was identified as *Corynebacterium diphtheriae* by VITEK® 2 ANC card (excellent identification 99%). This was further confirmed by MALDI-TOF MS (bioMérieux SA, Marcy-l'Etoile, France), with a confidence value of 99.9.

The case was reported on day 7 to the Department of Epidemiological Surveillance and Intervention of the Greek National Public Health Organization (NPHO) through the Mandatory Notification System.

The isolate was sent to the WHO Collaborating Centre for Reference and Research on Diphtheria and Streptococcal Infections housed under the auspices of Public Health England at Colindale, UK for confirmation of identification and the determination of toxigenicity. These were achieved using a combination of phenotypic methods and real-time PCR [22]. The analysis confirmed *C. diphtheriae* of the mitis biovar and the presence of the diphtheria toxin gene. The modified Elek immunoprecipitation test [23] was used to confirm the production of diphtheria toxin. Multi-locus sequence typing (MLST) profiles [24] were derived from whole-genome sequencing using metric-oriented sequence typing [25,26]. Alleles, allelic profiles and sequence types (STs) were determined by comparison with the *Corynebacterium diphtheriae* MLST Database (https://pubmlst.org/cdiphtheriae/ accessioned 18 December 2019). The genotype was identified as a novel ST (allelic profile 3-10-3-1-3-3-2) which was a single locus variant (SLV) of ST-574.

The patient's immunological response was investigated retrospectively (Table 1). Immunoglobulins were measured by nephelometry (Dade Behring –Siemens) and immunoglobulin G (IgG) levels were found to be low for his age. Antibody response tests for tetanus toxoid and anti-pneumococcal capsular polysaccharide (Anti-PCP) were performed in the Department of Immunology – Histocompatibility of ‘Aghia Sophia’ Children's Hospital in Athens. In specific, VaccZyme Anti-Tetanus Toxoid IgG Enzyme Immunoassay kit and VaccZyme Anti-PCP IgG Enzyme Immunoassay kit (The Binding Site Group Ltd) were used. In addition, indirect chemiluminescent immunoassay to test quantitatively IgG antibodies against Corynobacterium diphtheria toxin in human serum, in order to determine the protective status (Virclia, Vircell Granada, Spain) was performed in ‘P. & A. Kyriakou’ Children's Hospital. Anti-HBs and IgG for rubella were measured using a chemiluminescence assay with the Architect i2000SR (Abbott, Illinois, USA). Concerning the above pathogens and according to the Greek National Immunization Programme (NIP) the boy was incompletely vaccinated for tetanus and diphtheria (three doses instead of five), *S*treptococcus *pneumoniae* (two doses instead of four) and measles–mumps–rubella (one dose instead of two), but fully vaccinated against Hepatitis B virus (HBV). However, although fully vaccinated for HBV he showed no antibody response. In addition, along with incomplete vaccination for tetanus, diphtheria, pneumococcus and rubella his antibody response was insufficient to provide protection from infection. The above findings are indicative of primary immunodeficiency and specifically fulfil the diagnostic criteria of antibody deficiency because of his inability to produce specific antibodies.

**Table 1.** Immunological parameters of the index case tested retrospectively

Immunological parameters	Patient's results	Normal range or protective levels
IgG	418 mg/dL	NR: 891–2042 mg/dL
IgA	90.7 mg/dL	NR: 52–331 mg/dL
IgM	59.1 mg/dL	NR: 63–275 mg/dL
Abs anti-tetanus toxoid IgG	0.1 IU/mL	PL: >0.1 IU/mL^a^
Abs anti-diphtheria toxoid IgG	0.01 IU/mL	PL: >0.1 IU/mL^a^
Abs anti-PCP IgG	5 mg/L	PL: >60 mg/L
IgG for rubella	3.8 IU/mL	PL: ⩾5 IU/mL
Anti-HBs	0.99 mIU/mL	PL: ⩾10 mIU/mL

Abs, antibodies; Anti-HBs, antibodies to the Hepatitis B surface antigen; NR, normal range; PCP, pneumococcal capsular polysaccharide; PL, protective level after vaccination.

a For diphtheria and tetanus, titres of 0.01 to <0.1 IU/mL are considered to provide partial protection.

### Public health response

The epidemiological investigation was conducted by the NPHO in cooperation with the relevant Directorate of Public Health and was based on the new WHO Surveillance standards for diphtheria [21]. According to the European Commission's case definition as amended in 2012 a confirmed case of respiratory diphtheria is any person with the clinical form of classic or mild respiratory diphtheria (an upper respiratory tract illness with laryngitis or nasopharyngitis or tonsillitis and/or without an adherent membrane/pseudomembrane, respectively) from whom a diphtheria toxin-producing *C. diphtheriae*, *C. ulcerans* or *C. pseudotuberculosis* has been isolated from a clinical specimen. A probable case is defined as any person meeting the clinical criteria for classic or mild respiratory diphtheria with an epidemiological link to a human confirmed case or an animal to human transmission. A suspected case is defined as any person meeting the clinical criteria for classic respiratory diphtheria.

The boy had no siblings and lived with his mother. Based on the vaccines registered in his health booklet the child had been immunised with three doses of a DTaP vaccine during the first 2 years of life. There was no travel history or any particular contact with people who have recently travelled to an endemic country. During the week before the symptoms’ onset, the child was attending a primary school with 17 pupils in his class.

As soon as the case was notified, the epidemiological investigation was initiated by the NPHO aiming to identify all close contacts within the family and the school environment as well as in the hospital where the child was treated and implement all necessary measures according to the WHO protocol [21].

**Fig. 1.**
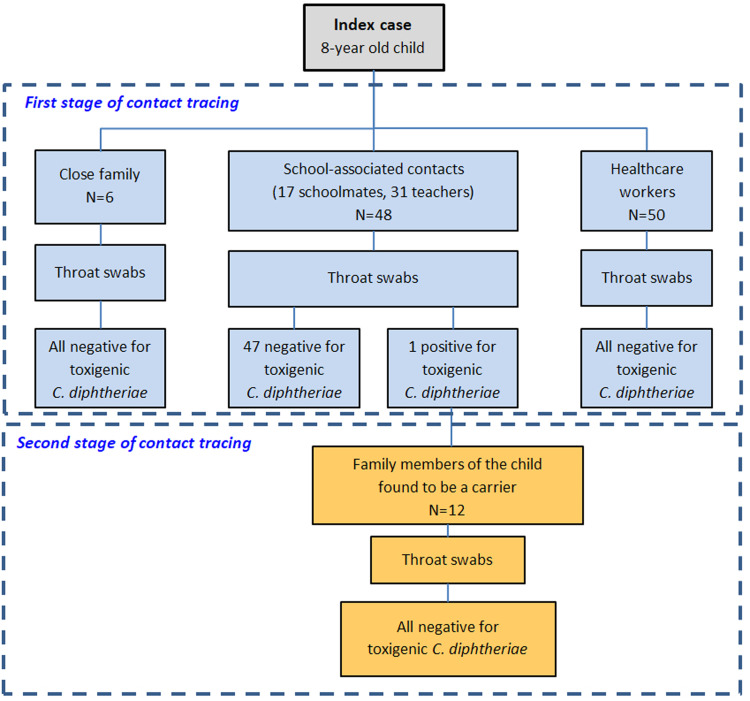
The two stages of contact tracing during the epidemiological investigation of the index case.

Contact tracing was carried out in two phases (Fig. 1). In the first phase, close contacts of the index case were identified. Those included a total of 104 individuals: six people from the family environment (mother and relatives), 48 people from the school environment (classmates, teachers and other school servants) and 50 healthcare workers from the two hospitals that had been potentially exposed to the child's respiratory secretions. All contacts were examined for the existence of classic respiratory diphtheria symptoms and a pharyngeal swab specimen was collected from each one of them. Prophylactic clarithromycin (30 mg/kg orally daily for the children and 1 g orally daily for adults) was given to all close contacts for a period of seven days and a daily check-up for the presence of new clinical symptoms was recommended. In addition, the vaccination status of all contacts was checked. Among the 17 children, 15 were completely vaccinated for their age with five doses of DTaP and two were incompletely vaccinated, one with four doses and the other with three doses of DTaP. The vaccination status of adult contacts was either unknown or impossible to be certified and thus they were all recommended to get a booster dose of Tdap from their treating physicians.

The 54 pharyngeal specimens from the family and school contact tracing were examined at the Central Public Health Laboratory while 50 specimens from the potentially exposed health-care workers were examined at the laboratory of clinical microbiology of ‘P. and A. Kyriakou’ Children's Hospital with microbiological culture on standard blood agar and serum tellurite agar (Bioprepare, Greece) and incubation at 35 ± 2 °C for 24–72 h with subsequent Gram stain of all suspicious colonies. Further identification of catalase-positive, Gram-positive coryneforms was performed by conventional biochemical testing with API® Coryne or VITEK® 2 ANC (bioMérieux SA, Marcy-l'Etoile, France). *C. diphtheriae* was identified from one suspect isolate as *C. diphtheriae* biovar *mitis*. The identification was confirmed with MALDI-TOF MS and a pure subculture of the isolate was sent to the WHO Collaborating Centre for Reference and Research on Diphtheria and Streptococcal Infections, Public Health England, UK for confirmation of identification and the determination of toxigenicity (as described above). The analysis confirmed a toxigenic strain of *C. diphtheriae* biovar mitis. Furthermore, the genotype was characterised as the same novel ST (allelic profile 3-10-3-1-3-3-2) as the index case. This novel profile was subsequently submitted to the *C. diphtheriae* MLST database and designated ST698 by the curators.

The single laboratory-confirmed case of toxigenic *C. diphtheriae* amongst the screened contacts of the index case was an asymptomatic carrier. This was a 9-year-old boy, a classmate of the index case, who belonged to a special subpopulation group (Roma). The child was inadequately vaccinated with three doses of a DTaP vaccine. A second stage of contact tracing took place through which 12 family members (six children and six adults) were identified as close contacts. Neither the asymptomatic carrier nor any of his close contacts had a history of travel to an endemic country. Pharyngeal swabs were collected from all contacts following the same protocol and all tested negative. Oral clarithromycin (30 mg/kg per day for children and 1 g per day for adults) was administered as chemoprophylaxis to the carrier for 14 days and all the contacts for 7 days. A second pharyngeal swab specimen was obtained from the carrier after concluding the treatment which was found negative. Booster doses of DTaP and Tdap were given to children and adult contacts based on available data regarding their vaccination status. No further cases occurred during the prospective follow-up of exposed individuals.

## Discussion

The epidemiology of diphtheria in Greece, as in other western European countries, has changed dramatically in the last 60 years. The introduction of systematic immunisation (monovalent diphtheria vaccine was introduced in the Greek NIP in 1951 while DTP in 1961) along with the increase of the standard of living and the level of health services led to a significant reduction of morbidity. Before 1960 the disease was endemic in Greece, but since then the average annual incidence gradually reached 5.4 per 100 000 population in the period 1967–1977 and in the 1980s reached zero. It has been over 35 years since the last autochthonous case of diphtheria has been identified in Greece (1982) while the country has remained diphtheria free since 1994 (last three imported cases during 1992–1994). The report of a diphtheria case triggered the immediate response from the NPHO and the coordination of other public health authorities. Through coordinated efforts, contacts were traced and evaluated for diphtheria symptoms and in addition to carriage; antibiotic chemoprophylaxis was offered as well as vaccination to complete the immunisation status of all individuals. The whole process has put to the test the national surveillance, laboratory capacity and response system regarding the potential for diphtheria re-emergence and offered valuable insights for its future optimisation.

The atypical clinical presentation that was reminiscent of lower respiratory tract infection (laryngotracheitis and or pneumonia) with only minor patches of exudate that did not develop into a typical membrane confounded the diagnosis. It has been previously documented that the minor involvement of the pharynx can usually lead to either missed or delayed diagnosis and that vaccinated, or partially vaccinated individuals may not develop a pseudomembrane [15,27]. In particular, a quarter of all cases of laryngeal diphtheria do not present with a pharyngeal lesion. However, although laryngeal diphtheria is rare (25% of respiratory cases) it is associated with greater morbidity and mortality [3]. A similar case of laryngeal diphtheria with a fatal outcome was reported in an unimmunised child in the UK in 2008 [28]. Nowadays, diphtheria cases in Europe are uncommon and vaccination coverage in most countries is high, thus leading to milder and atypical clinical manifestations [17]. Furthermore, the decreased incidence of the disease has led to decreasing clinical experience; thus, the diagnosis becomes a greater challenge.

This case was not an unexpected event and it is not unique, even for non-endemic European countries. Similar cases have been described recently in unvaccinated children with a fatal outcome; one in a 6-year-old child in Spain [29] and the other in a 3-year old child in Belgium [17].

Asymptomatic respiratory carriers within vaccinated individuals remain an important route of transmission of diphtheria and a public health problem, especially for vulnerable population groups with incomplete vaccination or immunosuppression as in our case.

Immunodeficiency has been reported in patients with DS including: mild to moderate T- and B-cell lymphopenia, with a marked decrease of naive lymphocytes, impaired mitogen-induced T-cell proliferation, suboptimal antibody responses to immunisations and defects of neutrophil chemotaxis. In particular, in children with DS, specific antibody responses are elicited but often in lower titres than in non-DS controls which are in accordance with the increased frequency of respiratory tract infections [30].

Vaccination with DTP/DTaP is among the most implemented in the Greek NIP. The current NIP includes five doses of DTaP: the initial three are administered at 2, 4 and 6 months of age, the fourth at the age of 15–18 months and the fifth at the age of 4–6 years. A dose of Tdap is administered at the age of 11–12 years. Subsequently, a booster dose of Td/Tdap is recommended every 10 years by the NIP. According to the last national vaccination coverage study performed in 2012 in first-grade school children, 98.9% and 89.5% were found vaccinated with four doses and five doses of diphtheria containing vaccine, respectively [31]. A similarly high percentage of children attending kindergarten vaccinated with four doses of DTaP (95.8%) was recorded in another national study in 2014 [32]. However, both studies recorded a slight delay in the timely vaccination with four doses by the age of 24 months (82.9% in 2012 and 85.3% in 2014) and with five doses by the age of 6 years (83.2% in 2012). Of particular importance is the low vaccination coverage of Roma children with four doses, ranging between 28 and 31%, which was documented in the national study of 2012 emphasising the significance of pockets of suboptimally vaccinated population groups [31]. In addition, there were no specific data regarding the vaccination coverage of adults. A multinational study recorded a decline in recently vaccinated participants from Greece with increasing age (*x*^2^(3) = 7.925, *p* = 0.048; 35–44 years: 15%; 45–54 years: 13%; 55–64 years: 7%; 65–74 years: 5%) showing a diminishing adherence in receiving the booster dose of Td every 10 years as recommended by the NIP [33].

Moreover, the increased flow of immigrants and asylum seekers in Greece, especially from endemic countries where the health systems have collapsed, poses an additional risk of outbreaks in these populations. Such populations are usually incompletely vaccinated, travel under adverse conditions, or live for a period in detention centres exposed to over-crowding and poor hygiene, thus increasing the risk of diphtheria. In the past, this has led to the report of several cases of cutaneous diphtheria in some European countries [34,35]. The vaccination status of such populations needs to be checked and catch-up vaccinations should be offered at any opportunity [1]. Moreover, physicians at the points of entry must be alerted to have the appropriate level of clinical suspicion in order to early detect potential cutaneous diphtheria cases.

There are some important laboratory issues that concern the rarity of cases and the expense and complexity associated with the laboratory diagnosis of *C. diphtheriae* that has led many countries in Europe, including Greece, to cease the systematic screening of throat specimens; therefore, expertise and recognition of the organism declined [10,36]. This decline was captured by a gap analysis of diphtheria diagnostics among the Member States, commissioned by European Centre for Disease Prevention and Control (ECDC) in 2017, in order to collect systematic information about laboratory capacity related to diphtheria control. This gap analysis demonstrated that there are significant gaps in diphtheria diagnostic capacity within the EU/EEA, with only six out of 30 Member States fulfilling the minimum criteria in terms of surveillance, specialised laboratory diagnostics and expertise [37].

Greece has only partial diagnostic capacity for diphtheria since a national reference laboratory, with the capacity to test for the diphtheria toxin gene by PCR and/or expression using the Elek-test, has not yet been established. Therefore, it is essential that the country's diagnostic capability be enhanced to detect toxigenicity and undertake molecular characterisation of all three potentially toxigenic corynebacteria – *C. diphtheriae*, *C. ulcerans* and *C. pseudotuberculosis*.

The molecular characterisation of the two *C. diphtheriae* isolates undertaken by the WHO Collaborating Centre for Reference and Research on Diphtheria and Streptococcal Infections revealed that both belonged to the same, novel MLST type (ST698), a SLV closest to ST574 which was originally isolated from a diphtheria case in India. MLST provides a valuable tool for monitoring and characterising endemic and epidemic *C. diphtheriae* strains [23].

Nowadays, awareness of this potentially re-emerging disease relies mostly upon the collection of data through specific seroepidemiological or screening studies. A multinational study in 10 European countries revealed carriage rates of 0.8 per 1000 (95% confidence interval (CI) 0.1–2.9) and 0.7 (95% CI 0.1–2.4) for toxigenic *C. diphtheriae* strains in Latvia and Lithuania, respectively, while it was 0 in all other countries, but with upper 95% CI ranges varying from 0.4 per 1000 in the UK to 3.8 per 1000 in Italy [26]. In the most recent screening study from Greece, carried out in 2008, neither toxigenic *C. diphtheriae* nor *C. ulcerans* was identified among 2317 pharyngeal swabs from both children and adults from the general Greek population [38]. However, a study of 2100 serum samples from adults >35 years old in six European countries (Austria, Belgium, Germany, Greece, Italy and Poland) showed the lowest diphtheria-specific antibody concentration in Italy and Greece, leaving a considerable percentage of them unprotected (63 and 59%, respectively, below the protective concentrations of 0.1 IU/mL) [32].

This fatal case of diphtheria highlights the need for maintaining high vaccination coverage with both primary and booster doses for children and adults, as immunity weakens over time.

The production of DAT for export has either ceased or has been greatly reduced in the very few countries where it is still produced. Thus, although included in the WHO list of ‘essential medicines’ DAT is in short supply worldwide. The problem has been highlighted by scientists in many European countries and the ECDC and timely actions to secure availability of stock in countries have been recommended [39,40]. In our case, the diagnosis of diphtheria was made late in the disease course and was confirmed only after post mortem. It was only after the incident that a small quantity of DAT was secured from the WHO in order to respond to any additional cases of the disease.

Our report along with others in Spain and Belgium highlights the risk of unvaccinated individuals and especially vulnerable populations in non-endemic countries with high vaccination coverage to contract *C. diphtheriae* from asymptomatic carriers and develop the disease. Hence, maintaining a high level of epidemiological and microbiological surveillance, implementing systematic vaccination programmes with basic and booster doses in children, adults and especially vulnerable subpopulation groups, enhancing awareness amongst healthcare professionals, assuring the availability of DAT stocks to allow timely delivery to the patient are the cornerstones to prevent similar incidents in the future.

## Data Availability

The data that support the findings of this study are available from the corresponding author upon reasonable request.
